# Neuroglobin Regulates Wnt/β-Catenin and NFκB Signaling Pathway through Dvl1

**DOI:** 10.3390/ijms19072133

**Published:** 2018-07-23

**Authors:** Yu Xun, Zhen Li, Yingxin Tang, Manjun Yang, Shengwen Long, Pan Shu, Jiabing Li, Ye Xiao, Fen Tang, Chenxi Wei, Ning Liu, Shuanglin Xiang

**Affiliations:** 1 Key Laboratory of Protein Chemistry and Development Biology of State Education Ministry of China, College of Life Sciences, Hunan Normal University, Changsha 410081, Hunan, China; xunyu2@126.com (Y.X.); lizhen235@163.com (Z.L.); tangtang94@126.com (Y.T.); yangmanjun1205@gmail.com (M.Y.); m15595719565_1@163.com (S.L.); m15111060065_1@163.com (P.S.); ljb2406297246@163.com (J.L.); xy5488@163.com (Y.X.); frawn59@126.com (F.T.); weicx@hunnu.edu.cn (C.W.); 2 College of Medicine, Hunan Normal University, Changsha 410013, Hunan, China

**Keywords:** Ngb, Dvl1, NFκB, Wnt/β-Catenin, SK-N-SH

## Abstract

Neuroglobin is an endogenous neuroprotective protein, but the underlying neuroprotective mechanisms remain to be elucidated. Our previous yeast two-hybrid screening study identified that Dishevelled-1, a key hub protein of Wnt/β-Catenin signaling, is an interaction partner of Neuroglobin. In this study, we further examined the role of Neuroglobin in regulating Dishevelled-1 and the downstream Wnt/β-Catenin and NFκB signaling pathway. We found that Neuroglobin directly interacts with Dishevelled-1 by co-immunoprecipitation, and the two proteins are co-localized in both cytoplasma and nucleus of SK-N-SH cells. Moreover, the ectopic expression of Neuroglobin promotes the degradation of exogenous and endogenous Dishevelled-1 through the proteasomal degradation pathway. Furthermore, our results showed that Neuroglobin significantly inhibits the luciferase activity of Topflash reporter and the expression of β-Catenin mediated by Dishevelled-1 in SK-N-SH cells. In addition, we also documented that Neuroglobin enhances TNF-α-induced NFκB activation via down-regulating Dishevelled-1. Finally, 3-(4,5-Dimethylthiazol-2-Yl)-2,5-Diphenyltetrazolium Bromide (MTT) assays showed that Neuroglobin is an important neuroprotectant that protects SK-N-SH cells from TNF-α-induced decrease in cell viability. Taken together, these findings demonstrated that Neuroglobin functions as an important modulator of the Wnt/β-Catenin and NFκB signaling pathway through regulating Dishevelled-1.

## 1. Introduction

Neuroglobin (Ngb) has been first identified in 2000 as a novel oxygen-binding globin protein which is predominantly expressed in brain neurons and some endocrine tissues [1]. Numerous data suggested Ngb is an endogenous neuroprotectant in the brain, as increased Ngb expression inversely correlates with the severity of functional and histological deficits after focal cerebral ischemia [2], traumatic brain injury [3,4], and Alzheimer’s disease [5]. The partial mechanisms of Ngb neuroprotection include scavenging of reactive oxygen species (ROS) and sensing oxygen (O_2_) [6], preservation of mitochondria function [7], maintenance of the integrity of the cytoskeleton [8], and inhibition of apoptosis [9]. However, the molecular mechanisms of Ngb neuroprotection have not been fully elucidated.

Accumulating evidence suggested that Ngb may act as a signal transducer, and play a neuroprotective role by regulating multiple signaling pathways [10]. For example, Ngb had been found to regulate receptor-mediated G protein signaling by binding to the GDP-bound state of G protein G subunit (Gα) as a GDP-dissociation inhibitor (GDI) [11], and Ngb could negatively regulate Rho-GDI-GTPase signaling by interacting with Rac1 and RhoA, two key members of the Rho GTPase family [12]. Moreover, Ngb had been demonstrated to interact with PTEN (gene of phosphate and tension homology deleted on chromsome ten) and Akt [13], exerts neuroprotection via activating Akt signaling [14,15,16]. These studies suggested that Ngb may play a neuroprotective role in regulating multiple cell survival signaling pathways. Several papers reported the interacting partners of Ngb. Using the surface plasmon resonance technique, cystatin C and prion protein were identified as Ngb interactors [17,18,19]. It was proven that 14-3-3, cytochrome c and huntingtin can interact with Ngb via co-immunoprecipitation (IP) experiment [17,20,21]. Our previous study used a yeast two-hybrid system to screen a mouse brain cDNA library-identified Dvl1, an important hub protein of the canonical Wnt/β-Catenin signaling pathway, as a Ngb interaction protein [22], implying that Ngb may exert neuroprotective role by regulating Wnt/β-Catenin signaling pathway. 

In this study, to further elucidate the molecular mechanisms of Ngb neuroprotection, we investigated the interaction between Ngb and Dvl1, and clarified the roles of Ngb in regulating Dvl1 as well as the Wnt/β-Catenin signaling pathway. Because Dvl1 protein have been reported to interact with p65 and acts as a repressor of NFκB signaling pathway [23], we also investigated whether Ngb is involved in regulating TNF-α-mediated NFκB signaling pathway via binding to Dvl1. Moreover, we determined the effect of Ngb and Dvl1 on TNF-α-induced decrease in cell viability of SK-N-SH cells. Our results demonstrated that Ngb directly interacts with Dvl1, and facilitates the degradation of Dvl1 via ubiquitin-proteasome pathway, and thereby inhibits Wnt/β-Catenin and increases NFκB signaling pathway, respectively. Finally, our study showed that Ngb knockdown and overexpression exacerbates and attenuates TNF-α-induced decrease in cell viability of SK-N-SH cells, respectively.

## 2. Results

### 2.1. Ngb Interacts with Dvl1

**Figure 1:**
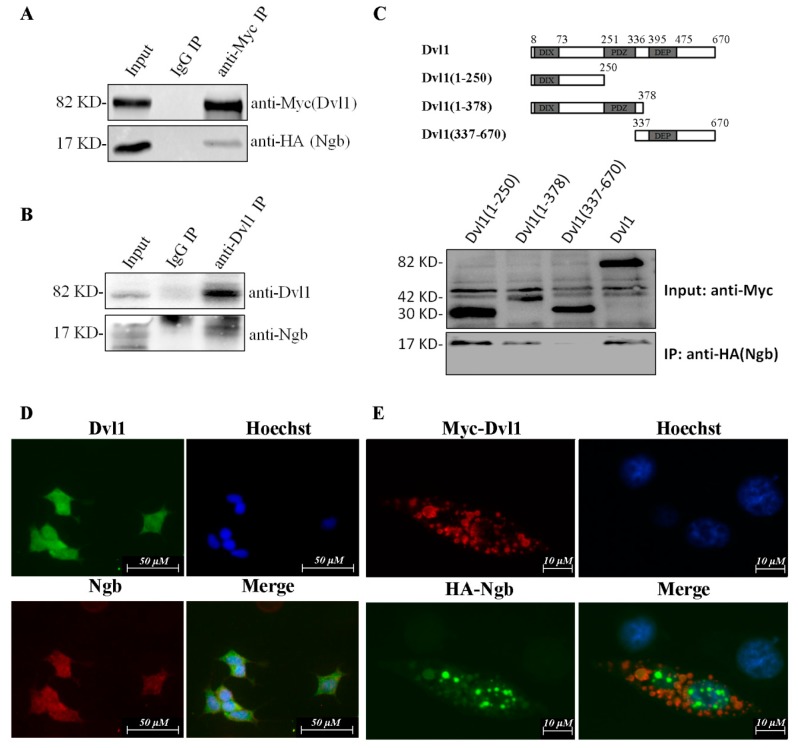
Neuroglobin (Ngb) interacts with Dvl1 in SKNSH cells. (**A**) HA-Ngb was co-transfected with Myc-Dvl1 in SK-N-SH cells. Cells were harvested after 24 h post-transfection. Cells extract (600 μg) were precipitated by anti-Myc rabbit polyclonal antibody or control Immunoglobulin G (IgG). Western blot was used to detect Myc-Dvl1 and HA-Ngb. (**B**) Cells extract (600 μg) were prepared and immunoprecipitation (IP) with rabbit anti-Dvl1 antibody or control rabbit IgG. Western blot was used to detect Dvl1 and Ngb. (**C**) Dvl1(1-250), Dvl1(1-378), Dvl1(337-670) or Myc-Dvl1 was co-transfected with HA-Ngb. After 24 h post-transfection, cells were harvested and IP with mouse anti-Myc monoclonal antibody. Western blot was used to detect Ngb. (**D**) SK-N-SH cells were seeded in 6-well plate. The mouse anti-Ngb monoclonal antibody and Texas Red-conjugated anti-mouse IgG (red) were used to detect Ngb protein. The rabbit anti-Dvl1 monoclonal antibody and Texas Green-conjugated anti-rabbit IgG were used to detect Dvl1 protein. Nuclei were stained by Hoechst 33258. The merged image showed the co-localization of Dvl1 and Ngb. (**E**) SK-N-SH cells were co-transfected with Myc-Dvl1 and HA-Ngb. The rabbit anti-HA polyclonal antibody and Texas Green-conjugated anti-rabbit IgG were used to detect HA-Ngb protein. The mouse anti-Myc monoclonal antibody and Texas Red-conjugated anti-rabbit IgG were used to detect Myc-Dvl1 protein. Nuclei were stained by Hoechst 33258. The merged image showed the co-localization of Myc-Dvl1 and HA-Ngb.

Our previous results of yeast-two-hybrid screening using a mouse embryonic cDNA library identified that mouse Dvl1 could potentially interact with Ngb [22]; we further performed co-immunoprecipitation (IP) experiments to confirm whether Ngb interacts with Dvl1 in human SK-N-SH cells. We co-transfected Myc-Dvl1 and HA-Ngb into SK-N-SH cells. At 24 h post-transfection, cells were harvested, and cell lysates were precipitated by anti-Myc rabbit polyclonal antibody or control Immunoglobulin g (IgG). Anti-HA mouse monoclonal antibody was used to detect the presence of Myc-Dvl1 in the precipitated complex. As shown in Figure 1A, HA-Ngb can be precipitated by Myc-Dvl1. Our data also showed that endogenous Ngb can be precipitated by anti-Dvl1 antibody (Figure 1B). Then, we mapped the Ngb-binding domain in Dvl1. Three truncation mutants of Dvl-1 were cloned into the pCMV-Myc vector. Myc-Dvl1(1–250) contains DIX domain which is key for both PCP and β-catenin signaling [24]. Myc-Dvl1(1–378) contains both DIX and PDZ domain. The PDZ mediates curial protein–protein interactions and is important for both canonical and non-canonical Wnt pathway [25]. Myc-Dvl1(337–670) only contains DEP domain which is curial for interaction with DAAM1 and activation of non-canonical signaling pathway [26]. Then Myc-Dvl1 (1–250), Myc-Dvl1(1–378) or Myc-Dvl1(337–670) co-transfected with HA-Ngb into SK-N-SH cells, respectively. Co-IP experiments showed that Ngb has stronger interaction with Myc-Dvl1(1–250), compared to other two truncation mutants (Figure 1C). Myc-Dvl1 (337–670), which only contains DEP domain, has no interactions with Ngb.

To further determine the localization of Ngb and Dvl1, immunocytochemistry was performed. We found that Ngb co-localized with Dvl1 in both cytoplasma and nucleus of SK-N-SH (Figure 1D). To better observe the location of Dvl1 and Ngb, Myc-Dvl1 and HA-Ngb were co-transfected into SK-N-SH cells; the mouse anti-Myc monoclonal antibody combined with Texas Red-conjugated anti-mouse IgG (red) were used to detect Myc-Dvl1 protein. The rabbit anti-HA monoclonal antibody and Texas Green-conjugated anti-rabbit IgG were used to detect HA-Ngb protein. As shown in Figure 1E, most of exogenous Dvl1 and Ngb were co-localized in cytoplasma, and a small part of exogenous Dvl1 and Ngb were co-localized in nucleus. These findings strongly supported that Ngb interacts with Dvl1.

### 2.2. Ngb Promotes the Proteasomal Degradation of Dvl1

**Figure 2:**
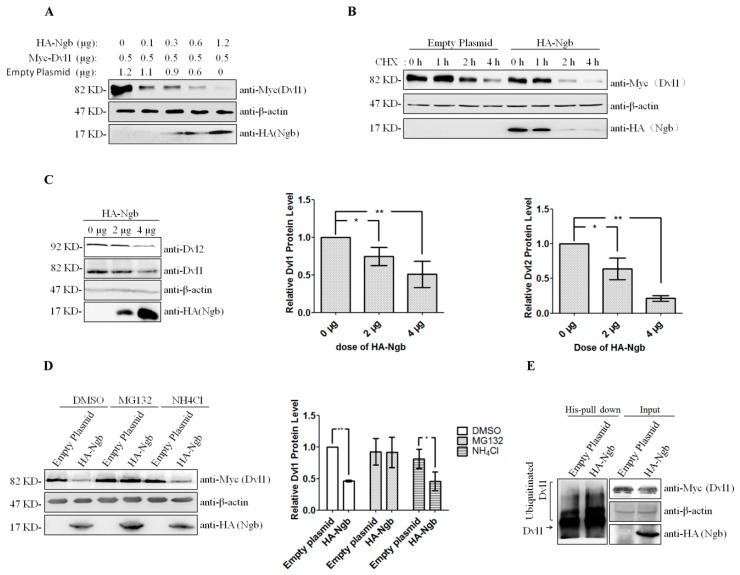
Ngb promotes the proteasomal degradation of Dvl1. (**A**) SK-N-SH cells were seeded in 6-well plate and transfected with plasmids as described. Cells were harvested after 24 h post-transfection. (**B**) SK-N-SH cells were cultured in 6 cm plate and transfected with the increased amount of HA-Ngb as described. Western blot was used to detect the expression of Dvl1 and Dvl2. densitometric analysis of western blot showed the effect of Ngb on Dvl1 and Dvl2 protein levels. β-actin served as equal loading controls. (Mean ± SD). *n* = 3, * *p <* 0.05, ** *p <* 0.01 versus cells transfected with empty plasmid. (**C**) 2 μg HA-Ngb or empty plasmid (pCMV-HA) was co-transfected with 0.5 μg Myc-Dvl1. After 12 h of post-transfection, cells were treated with CHX for 0, 1, 2, and 4 h. (**D**) Cells were treated with DMSO, MG132, or NH_4_Cl for 8 h. Densitometric analysis of western blot showed the effect of MG132 and NH_4_Cl on the Ngb-induced degradation of Dvl1β-actin served as equal loading controls. (Mean ± SD). *n* = 3, * *p* < 0.05, ** *p* < 0.01 versus cells transfected with controls as described. (**E**) His-Ub and Myc-Dvl1 were co-transfected with HA-Ngb or empty plasmid (pCMV-HA) into SK-N-SH cells. Then the His-ubiquitinated proteins were isolated from cell extracts using NTA agarose. Western blot was used to detect ubiquitinated Dvl1.

Our previous data indicated that Ngb binds to ubiquitin C (UBC) in yeast-two-hybrid screening system [17], implying that Ngb may potentially be associated with protein degradation through ubiquitin-proteasome pathway. To investigate whether Ngb regulates Dvl1 protein stability through their interaction, increasing amounts of HA-Ngb plasmids were co-transfected with a constant amount of Myc-Dvl1 plasmid in SK-N-SH cells, and western blot was used to detect the expression of exogenous Dvl1. As shown in Figure 2A, Ngb suppresses the expression of exogenous Dvl1 in a dose-dependent manner. Further, we also observed that Ngb overexpression significantly down-regulated endogenous Dvl1 and Dvl2 protein levels (Figure 2B).

To determine whether the decreased Dvl1 level resulted from protein degradation, SK-N-SH cells were co-transfected with Myc-Dvl1 plasmid and HA-Ngb plasmid or empty plasmid (pCMV-HA) for 12 h, followed by treatment with heximide (CHX), a protein translation inhibitor for another 0, 1, 2, or 4 h. The results showed that Myc-Dvl1 fusion protein degraded more rapidly in the cells transfected with HA-Ngb plasmid, compared to empty plasmid (Figure 2C), indicating that Ngb suppresses Dvl1 protein via a protein translation-independent manner. To further investigate the potential mechanisms underlying Ngb-induced Dvl1 protein degradation, Myc-Dvl1 plasmids were co-transfected with or without HA-Ngb plasmids into SK-N-SH cells for 12 h, followed by treatment with DMSO, proteasomal inhibitor MG132, and lysosomal inhibitor NH_4_Cl, respectively. The results showed that MG132 strongly suppresses Ngb-induced decrease of Dvl1 (Figure 2D). To further determine whether Ngb promotes ubiquitination of Dvl1, His-Ub and Myc-Dvl1 were co-transfected with or without HA-Ngb into SK-N-SH cells, and the His-Ub binding protein complex was isolated from cell extracts using NTA agarose. Western blot results showed that Ngb overexpression promotes Dvl1 polyubiquitination (Figure 2E). These results indicated that Ngb promotes the proteasomal degradation of Dvl1.

### 2.3. Ngb Inhibits Wnt/β-Catenin Signaling Pathway via Dvl1

**Figure 3:**
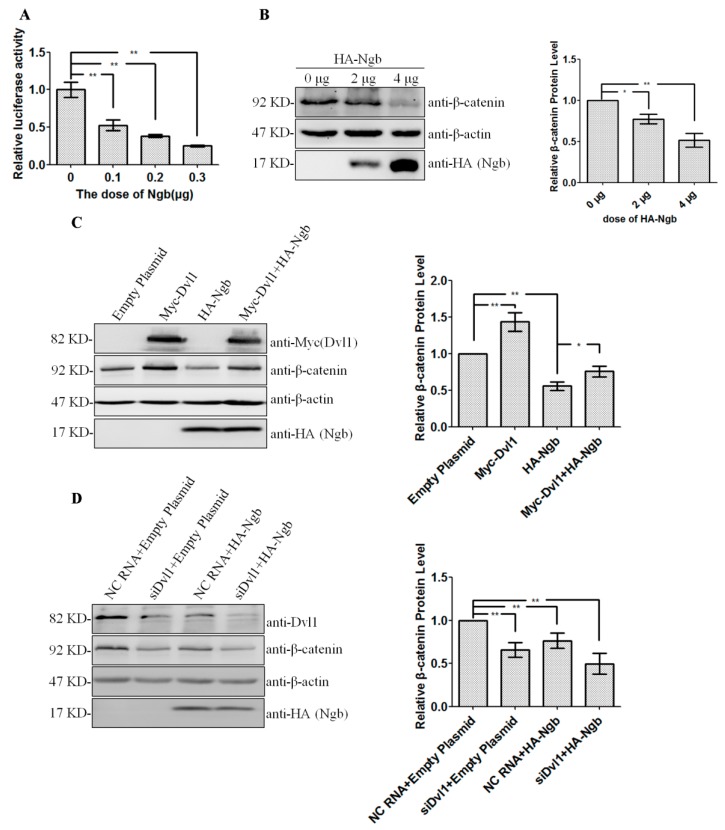
Ngb inhibits Wnt signaling pathway through decreasing Dvl1. (**A**) SK-N-SH cells were seeded on 24-well plates. Increased amount of HA-Ngb was co-transfected with constant amount of PRL-TK and pTOPFLASH. Luciferase activity (Mean ± SD) was measured after 24 h post-transfection. *n* = 3, ** *p* < 0.01 compared with controls. (**B**) Increased amount of HA-Ngb was transfected into SK-N-SH cells. The level of β-catenin proteins was detected by western blot. Densitometric analysis of western blot was performed. β-actin served as equal loading controls. (Mean ± SD). *n* = 3, ** *p <* 0.01 versus cells transfected with empty plasmid. (**C**) HA-Ngb or empty plasmid (pCMV-HA) was co-transfected with Myc-Dvl1 or empty plasmid (pCMV-Myc). Densitometric analysis of western bolt was performed. β-actin served as equal loading controls. (Mean ± SD). *n* = 3, * *p* < 0.05, ** *p* < 0.01 versus controls as described. (**D**) HA-Ngb or empty plasmid (pCMV-HA) was co-transfected with NC siRNA or Dvl1 siRNA. Western blot was performed after transfection for 48 h. Densitometric analysis of western bolt was performed. β-actin served as equal loading controls. (Mean ± SD). *n* = 3, ** *p* < 0.01 versus controls.

Dvl proteins are key upstream mediators of the Wnt/β-catenin signaling pathway [19]. To investigate whether Ngb down-regulates Dvl1, and subsequently inhibits Wnt/β-catenin signaling pathway, SK-N-SH cells were transiently transfected with increasing amounts of HA-Ngb plasmid with constant amounts of PRL-TK plasmid and pTOPFLASH plasmid. The PRL-TK plasmid contains a cDNA encoding Renilla luciferase and is usually used as internal control reporter. The pTOPFLASH plasmid is a luciferase reporter containing β-catenin binding sites [20]. Luciferase assay showed that Ngb overexpression strongly suppresses Wnt/β-catenin pathway in a dose-dependent manner (Figure 3A). To further confirm our hypothesis, increased amounts of HA-Ngb plasmid were transfected into SK-N-SH cells, and the β-catenin protein level was detected by Western blot. The results showed that Ngb overexpression can down-regulate the expression of β-catenin (Figure 3B). To determine whether Ngb-induced inhibition of Wnt/β-catenin pathway was mediated by Dvl1, SK-N-SH cells were transfected with Empty plasmid, HA-Ngb, Myc-Dvl1, or Myc-Dvl1 plus HA-Ngb plasmids. Western blot results showed that Dvl1 overexpression could rescue Ngb-induced down-regulation of β-catenin (Figure 3C). Moreover, the effect of Ngb overexpression on β-catenin protein level was also detected when the cells were also co-transfected with Dvl1 siRNA. The results showed that HA-Ngb and siDvl can attenuate β-catenin protein level, respectively, and Ngb can not further decrease β-catenin when silencing Dvl1 (Figure 3D). Taken together, these results suggest that Ngb inhibits the Wnt/β-catenin signaling pathway via regulating Dvl1.

### 2.4. Ngb Enhances TNF-α-Induced NFκB Activation via Down-Regulating Dvl1

**Figure 4:**
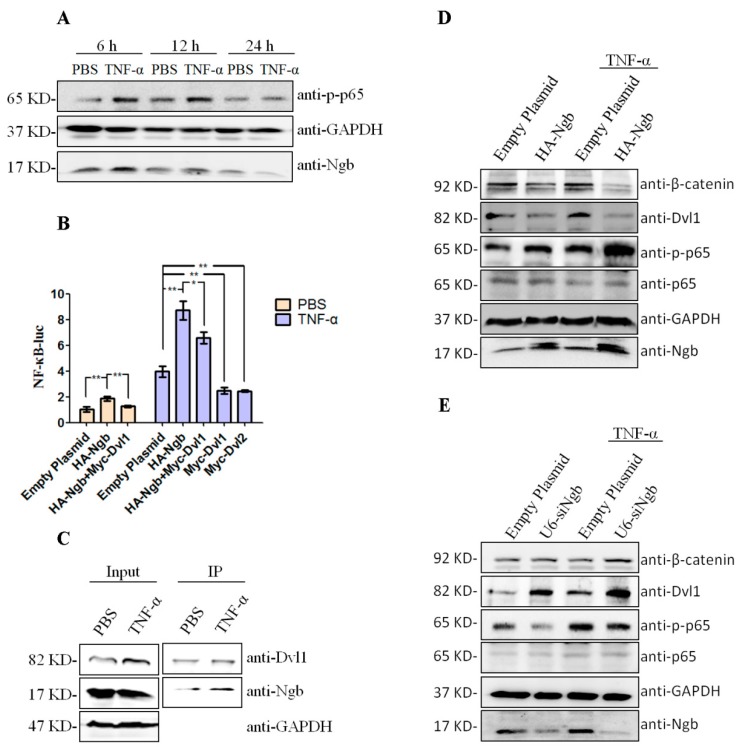
Ngb enhances TNF-α-induced-NFκB activation via down-regulating Dvl1. (**A**) SK-N-SH cells were treated with 10 ng/mL TNF-α or PBS for 6, 12, or 24 h. (**B**) The plasmids as described were co-transfected with NFκB report plasmid and PRL-TK plasmid in SK-N-SH cells. After transfection for 24 h, cells were treated with 10 ng/mL TNF-α or PBS for 6 h before Luciferase assays were performed. Data were presented as relative luciferase activities normalized to Renilla luciferase activities *n* = 4, * *p* < 0.05; ** *p* < 0.01 versus controls. (**C**) Cells were seeded in 10 cm plates and treated with 10 ng/mL TNF-α or PBS for 1 h. Cells’ lysates were immunoprecipitated with the anti-Dvl1 antibody. Western blot was used to detect Ngb and Dvl1. (**D**) HA-Ngb or empty plasmid (pCMV-HA) was transfected into SK-N-SH cells. After 24 h of post-transfection, cells were treated with TNF-α or PBS for 6 h. (**E**) U6-siNgb or empty plasmid (pRNAT-U6.1) was transfected into SK-N-SH cells. After 24 h of post-transfection, cells were treated with TNF-α or PBS for 6 h.

Our above data suggested that Ngb interacts with Dvl1, and previously, studies indicated that Dvls could inhibit TNF-α-induced NFκB activation and promote cell apoptosis in HEK 293 cells [23], implying that Ngb may be involved in regulating TNF-α-induced NFκB activation via interacting with Dvl1. To investigate the expression association of Ngb with NFκB (p65) in response to TNF-α stimulation, SK-N-SH cells were treated with TNF-α for 6, 12 and 24 h, respectively, and Ngb, p-p65 protein levels were measured by Western blot. The results showed that exposure of SK-N-SH cells to TNF-α for 6 and 12 h increased the expression of Ngb and p-p65, indicating that Ngb may be a positive regulator of NFκB signaling pathway (Figure 4A). Next, luciferase assay was used to investigate whether Ngb promotes TNF-α-induced NFκB activation. As expected, Ngb strongly promotes NFκB activity under resting and TNF-α stimulation condition (Figure 4B). Moreover, we observed that overexpression of Dvl1 or Dvl2 have the inhibitory effect on TNF-α-induced NFκB activation in SK-N-SH cells, which is in agreement with previous reports that Dvl1 and Dvl2 can significantly inhibit TNF-α-induced NFκB transcriptional activity [23]. Interestingly, it is worth noting that Ngb significantly attenuates the inhibitory effect of Dvl1 on NFκB activity under TNF-α stimulation condition (Figure 4B). To further investigate whether Ngb promotes TNF-α–induced NFκB activation via regulating Dvl1, we performed Co-IP for Ngb and Dvl1 in SK-N-SH cells under normal resting and TNF stimulation condition. Our data showed that the amount of precipitated Ngb was increased in TNF-α stimulation condition (Figure 4C).

To further confirm whether Ngb enhances TNF-α-induced NFκB activation via regulating Dvl1, Western blot was used to determine the effect of Ngb overexpression or suppression on NFκB (p65), NFκB (p65) phosphorylation, β-catenin, and Dvl1 expression. The results showed that Ngb overexpression markedly increases p-p65, along with decrease of Dvl1 in SK-N-SH cells (Figure 4D). Meanwhile, we also observed that Ngb suppression by specific siRNA attenuates p-p65, which is accompanied with increase of Dvl1 and β-catenin under resting and TNF-α stimulation conditions (Figure 4E). Taken together, these results indicated that Ngb enhances TNF-α-induced NFκB activation through down-regulating Dvl1.

### 2.5. Ngb Protects SK-N-SH Cells from TNF-α-Induced Death

**Figure 5:**
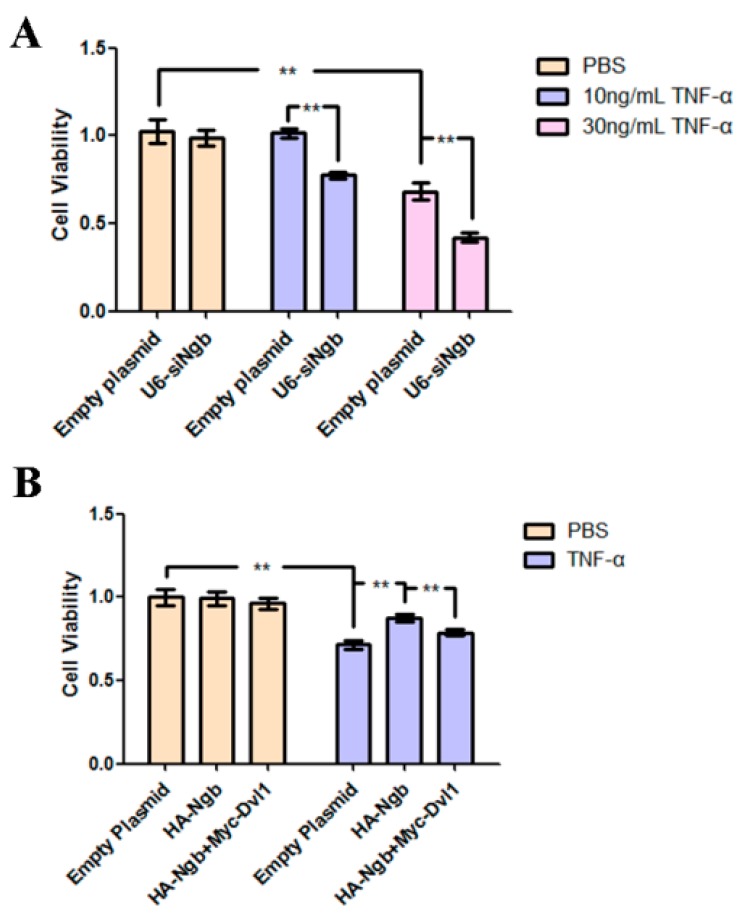
Ngb protects SK-N-SH cells from TNF-α-induced cell death. (**A**) SK-N-SH cells were transfected with empty plasmid (pRNAT-U6.1) or U6-siNgb. After 24 h post-transfection, cells were treated with PBS, 10 ng/mL or 30 ng/mL TNF-α for 24 h. 3-(4,5-Dimethylthiazol-2-Yl)-2,5-Diphenyltetrazolium Bromide (MTT) assay was used to measure cells’ viability. (Mean ± SD). *n* = 3, ** *p <* 0.01 versus controls. (**B**) Empty plasmid (pCMV-HA), HA-Ngb or HA-Ngb combined with Myc-Dvl1 was transfected into SK-N-SH cells. After 24 h of post-transfection, cells were treated with 30 ng/mL TNF-α for 24 h. Then MTT assay was used to measure cells viability. (Mean ± SD). *n* = 3, ** *p <* 0.01 versus controls.

Ngb increases NFκB activity via down-regulating the expression of Dvl1, which had been reported to promote cell death [23]. Here we examined the role of Ngb and Dvl1 in regulating TNF-α-induced decrease of cell viability. 3-(4,5-Dimethylthiazol-2-Yl)-2,5-Diphenyltetrazolium Bromide (MTT) assay showed that 10 ng/mL TNF-α treatment had no effect on cell viability SK-N-SH cells, but knockdown of endogenous Ngb by transfection with specific Ngb siRNA significantly decreased cell viability (Figure 5A). Moreover, we observed that Ngb suppression significantly exacerbated 30 ng/mL of TNF-α-reduced cell viability. Furthermore, treatment of empty plasmid-transfected SK-N-SH cells with 30 ng/mL of TNF-α for 24 h significantly decreases cell viability, and Ngb overexpression significantly attenuates TNF-α-reduced cell viability (Figure 5B). However, co-overexpression of Dvl1 significantly attenuated Ngb-mediated up-regulation of cells viability under TNF-α stimulation condition (Figure 5B). Therefore, these data indicated that Ngb attenuates TNF-α-induced decrease in cell viability of SK-N-SH cells.

## 3. Discussion

Although Ngb had been found to be involved in regulating multiple signaling pathways, including PI3K/Akt [14], Rho-GDI-GTPase [12], AMPK [27], and P38/GAP43 [28] signaling pathways, the roles of Ngb in regulating Wnt/β-Catenin and NFκB signaling pathways had rarely been reported. In this study, for the first time, we identified Dvl1 as a Ngb-binding protein, and showed that Ngb inhibits Wnt/β-Catenin and increases NFκB signaling pathway via regulating degradation of Dvl1 protein in human neuroblastoma SK-N-SH cells.

There are three Dishevelled homologues (Dvl1, 2, and 3) in human and mice, and Dvl had been considered as the hub of Wnt signaling pathway, including the canonical and non-canonical Wnt signaling pathway [29]. In the canonical Wnt signaling pathway, the Wnt ligands bind to its receptor Frizzled (Fz) and LRP5/6, subsequently resulting in the recruitment of Dvl to Fz and Axin, and dissociation of the β-catenin from a destruction complex composed of Axin, glycogen synthase kinase3β (GSK3β), adenmatous polyposis coli (APC) and casein kinase 1(CK1), and ultimately leading to the accumulation of β-catenin in nuclear and transcriptional activation of the Wnt target genes [30]. Therefore, Dvls are important regulatory points of Wnt signaling pathway. In this study, we found that Ngb was able to interact with Dvl1 N-terminal fragment (1–250), which contained the DIX domain. Interestingly, as a highly conserved region of three Dsh protein family members including Dvl1, 2, and 3, the DIX domain is very important for Dvls to form a complex with Axin and activate Wnt/β-Catenin [24]. It is worth noting that Ngb overexpression down-regulates the protein levels of Dvl2 (Figure 2B). Considering DIX domain is the Dsh homologous domain, Ngb may also interact with Dvl2 and Dvl3, and regulate their protein stability and function.

One more interesting finding is that Ngb promotes the degradation of Dvl1 via ubiquitin-proteasome pathway. However, the exact molecular mechanisms underlying Dvl1 degradation induced by Ngb still remain unknown. Although it had been rarely reported that Ngb was associated with protein degradation, our previous study by using yeast two-hybrid screening identified that Ngb potentially interacts with ubiquitin/proteasome-associated proteins including ubiquitin C, March7, Trim3, Ube2I3 and Zfp668 [22]. Among them, March7, Trim3, and Zfp668 are ubiquitin ligase. It is possible that Ngb promotes Dvl1 degradation via interacting with these ubiquitin ligases. This issue will be further investigated in the future.

The Wnt/β-Catenin signaling pathway participates in a wide range of biological processes, including embryonic development, oncogenesis [31]. It had also been reported that Wnt/β-Catenin signaling pathway plays crucial roles in neurogenesis [32] and neuronal differentiation [33]. As an essential effector of canonical Wnt/β-Catenin signaling pathway, Dvl1 is an important regulator in the proliferation and differentiation of neural progenitor cells [34]. Emerging data suggested that Ngb plays a key role in neurodevelopment and neurogenesis [35], and a most recent study showed that the loss of Ngb resulted in enhanced cell cycle progression and increased proliferation of neural stem cells [36]. However, the underlying molecular mechanisms still remain largely unknown. Our data found that Ngb is able to inhibit Wnt/β-Catenin signaling pathway via down-regulating Dvl1, which supports a potential role for Ngb in inhibiting proliferation of neural stem cells via regulating Wnt/β-Catenin signaling pathway. This issue will be further clarified in the future study.

The role of NFκB signaling pathway in determining neuronal cells’ fate is controversial. Activating NFκB was considered as a part of an inflammatory pathway leading to cell death [37,38,39]. However, accumulated evidence suggested that NFκB has neuroprotective function. Meffert et al. reported that p65 knockout mice suffered learning and memory problems [40]. Hippocampal neurons from p50 knockout mice were more sensitive to excitotoxic injury than non-transgenic mice [41]. Besides, Edward et al. reported p50 is important to protect neurons from ischemic injury [37]. Furthermore, inhibition of NFκB increased N2a cells death in OGD/R model [42]. So regulating NFκB signal pathway may become a new strategy to protect neural cells from apoptosis. We reported that Ngb positively regulates TNF-α induced NFκB activation via Dvl1. It is noteworthy that Dvl1 could inhibit NFκB activity in SK-N-SH cells under TNF-α stimulation condition, but Ngb could relieve Dvl1-mediated inhibition of NFκB. Moreover, we found that Ngb can enhance the activation of NFκB signaling induced by TNF-α via decreasing the expression of Dvl1. These findings were in agreement with a previous study that Dvls are repressor of TNF-α-stimulated NFκB activation [21]. Interestingly, Dvls had been found to interact with p65 in the nucleus and represses the DNA-binding activity of NFκB [21]. In the study, we observed that Ngb co-localized with Dvl1 in the both nucleus and cytoplasma of SK-N-SH cells (Figure 1D,E), and treatment of TNF-α for 1 h could not up-regulate Ngb protein levels, but markedly enhance the interaction of Ngb and Dvl1 (Figure 4C). These results implied that Ngb may directly interfere the binding of Dvl1 to p65 by competitively binding to Dvl1 in response to TNF-α stimulation, and thereby regulate NFκB activation.

Depending on the binding receptor and the intensity of stimulation, TNF-α had been found to exert a pro-apoptotic or an anti-apoptotic role [43]. In this study, we observed that treatment of empty plasmid-transfected SK-N-SH cells with 10 ng/mL of TNF-α for 24 h have no effect on cell viability, but knockdown of endogenous Ngb significantly resulted in decrease in cell viability. Moreover, suppression of endogenous Ngb dramatically exacerbated 10 ng/mL of TNF-α-induced decrease in cell viability of SK-N-SH cells (Figure 5A). It is possible that up-regulation of anti-apoptotic Ngb may be one of the endogenous neuroprotective mechanisms elicited by low dose or short time stimulation of TNF-α, but suppressed by high dose or long lasting stimulation of TNF-α. Of note, Ngb expression was up-regulated in SK-N-SH cells treated with 10 ng/mL of TNF-α for 6 and 12 h, but declined when treatment of TNF-α for 24 h (Figure 4A). Therefore, these findings indicated that Ngb expression alterations may be strongly associated with pathological stimulation, and Ngb is an important neuroprotectant under inflammatory conditions.

In conclusion, we found that Ngb is critically involved in regulation of Wnt/β-Catenin and NFκB signaling pathway via binding to Dvl1. We hope that our finding will not only lead to a better understanding of the role of Ngb in the physiological and pathological conditions, but also help in the development of Ngb-targeting therapeutic methods against various CNS disorders.

## 4. Materials and Methods

### 4.1. Materials

Full length human Dvl1, Dvl1(1–250), Dvl1(1–378) and Dvl1(337–670) was amplified by PCR from HeLa cDNA library using specific primers and inserted into pCMV-Myc (Clontech, Palo Alto, CA, USA). Full length human Ngb gene was amplified by PCR from SK-N-SH cDNA and inserted into pCMV-HA (Clontech, Palo Alto, CA, USA). The DNA fragments were designed according Ngb-targeted shRNA and were inserted into pRNAT-U6.1 (GenScript, Piscataway, NJ, USA). The TOPFlash report plasmid has been described previously [44]. The pLuc-NFκB vectors were purchased from Clontech (Palo Alto, CA, USA). The siDvl1 RNA was purchased from GenePharma (Shanghai, China) and the target sequence is 5-GGAGGAGATCTTTGATGACTT-3. The negative control siRNA was designed by GenePharma (Shanghai, China). Anti-Dvl1, anti-HA, anti-Myc and anti-p-NF-κB-p65 (ser 536) antibodies were purchased from Santa Cruz Biotechnology Inc (Santa Cruz, CA, USA), and anti-Dvl2 antibody was purchased from Cell Signaling Technology (Danvers, MA, USA). anti-Ngb primary antibody was obtained from BioVendor (Brno-Řečkovice a Mokrá Hora, Czech Republic). The primer information is shown in Table 1.

### 4.2. Cell Culture and Transfection

SK-N-SH cells were purchased from the Cell Bank of the Chinese Academy of Sciences (Shanghai, China) and cultured in DMEM (Gibco-BRL, Carlsbad, CA, USA) supplemented with 10% fetal bovine serum (Gibco-BRL, Carlsbad, CA, USA) and 100 μg/mL penicillin/streptomycin in a humidified (5% CO_2_, 37 °C) incubator. Plasmid DNA or siRNA was transfected into cells using LipofectamineTM 2000 (Invitrogen, Carlsbad, CA, USA) according to the manufacturer’s instructions.

### 4.3. Luciferase Reporter Assays

SK-N-SH cells with 70–80% confluence in 24-well plates were co-transfected with indicated plasmids as described in Figure 3A and Figure 4A and TOPFlash report plasmid or NFκB report plasmid. Luciferase activity was measured by Dual-Luciferase^®^ Reporter Assay System (Promega, Madison, WI, USA) in a TD-20/20 Luminometer (Turner Design, Sunnyvale, CA, USA). Renilla luciferase (PRL-TK) control plasmid was used to normalize the transfection efficiency.

### 4.4. Co-Immunoprecipitation and Western Blot

Co-immunoprecipitation (Co-IP) was performed as described previously [7]. Whole-cell lysed by RIPA buffer (50 Mm Tris–HCl (pH 7.2), 150 mM NaCl, 1% (*v*/*v*) Triton X-100, 1% (*w*/*v*) sodium deoxycholate, and 0.1% (*w*/*v*) SDS and protease inhibitors). Cell lysates were incubated with anti-Dvl1 rabbit polyclonal antibodies and Protein A/G plus-agarose (Santa Cruz) at 4 °C overnight. Cell lysates incubated with rabbit IgG were used as negative control. The immunoprecipitated proteins were separated by 15% SDS-PAGE gels and detected by anti-Ngb and anti-Dvl1 antibodies.

The anti-Tag IP assay is described below. SK-N-SH cells were co-transfected with Myc-Dvl1 and HA-Ngb. After 24 h post-transfection, cells were lysed by RIPA and incubated with anti-Myc rabbit polyclonal antibodies and Protein A/G plus-agarose at 4 °C overnight. Cells lysates incubated with rabbit IgG were used as negative control. The immunoprecipitated proteins were separated by 15% SDS-PAGE gels and detected by anti-HA and anti-Myc antibodies.

### 4.5. Immunocytochemistry

SK-N-SH cells were seeded on glass coverslips and cultured for 24 h. Then cells were fixed by 4% paraformaldehyde and co-incubated with rabbit polyclonal antibodies against Dvl1 and mouse polyclonal antibodies against Ngb for 4 h. Green-conjugated anti-rabbit IgG and Red-conjugated anti-mouse IgG (Thermo Fisher Scientific, Waltham, MA, USA) were added into cells and incubated for 2 h. Nucleus was stained with Hoechst 33258 (Sigma, St Louis, MO, USA). The signal was detected by fluorescence microscope (Zeiss, Oberkochen, Germany).

The immunocytochemistry assay for detecting the localization of exogenous Dvl1 and Ngb is described below. SK-N-SH cells were co-transfected with Myc-Dvl1 and HA-Ngb. After 24 h post-transfection, cells were fixed by 4% paraformaldehyde and co-incubated with rabbit polyclonal antibodies against HA-tag and mouse polyclonal antibodies against Myc-tag for 4 h. Green-conjugated anti-rabbit IgG and Red-conjugated anti-mouse IgG were added into cells and incubated for 2 h. Nucleus was stained with Hoechst 33258. The signal was detected by fluorescence microscope.

### 4.6. MTT Assays

2 × 10^5^ cells were seeded in 24 well plates. Subsequently, empty plasmids, HA-Ngb, Myc-Dvl1, HA-Ngb combined with Myc-Dvl1 or U6-siNgb were transfected into SK-N-SH cells for 24 h, followed by treatment with 10 or 30 ng/mL TNF-α for another 24 h. Cells were incubated with 1 mg/mL MTT for 4 h before 400 μL DMSO was added into each well. After formazan crystals completely dissolved, samples were measured at 570 nm using a spectrophotometer (UV-2102C, Double Kotewall, Hangzhou, China).

### 4.7. Statistical Analysis

Data were expressed as means ± S.D. from three to five independent experiments. Data was analyzed using student’s *t* test for two groups or ANOVA with Tukey–Kramer tests for multiple group comparisons. *p* < 0.05 was considered statistically significant.

## Figures and Tables

**Table 1:** Primers used in the present study.

Name	Orientation	Sequence (5′-3′)
HA-Ngb	Forward	ACGCGTCGACCATGGAGCGCCCGGAGCC
HA-Ngb	Reverse	ATAAGAATGCGGCCGTTACTCGCCATCCCAGCCTCG
Myc-Dvl1	Forward	ACGCGTCGACCATGGCGGAGACCAAGATTATCTA
Myc-Dvl1	Reverse	ATAAGAATGCGGCCGCTCACATGATGTCCACGAAGA
Dvl1(1-250)	Forward	ACGCGTCGACCATGGCGGAGACCAAGATTATCTA
Dvl1(1-250)	Reverse	ATAAGAAT GCGGCCGCTCAGACGATGTTGAGGGACATG
Dvl1(1-378)	Forward	ACGCGTCGACCATGGCGGAGACCAAGATTATCTA
Dvl1(1-378)	Reverse	ATAAGAATGCGGCCGCTCACTCGTAGCGGGGCAGG
Dvl1(337-670)	Forward	ACGCGTCGACCAAGTGCTGGGACCCAAC
Dvl1(337-670)	Reverse	ATAAGAATGCGGCCGCTCACATGATGTCCACGAAGA
U6-siNgb	Forward	GATCCGGTGATGCTCGTGATTGATGCTTCAAGAGAGCATCAATCACGAGCATCACCTTTTTTA
U6-siNgb	Reverse	AGCTTAAAAAAGGTGATGCTCGTGATTGATGCTCTCTTGAAGCATCAATCACGAGCATCACCG

## Abbreviations

Ngb	Neuroglobin
Dvl1	Dishevelled-1
Co-IP	co-immunoprecipitation
CHX	Heximide
TNF-α	tumor necrosis factor alpha
siRNA	small interfering RNA
NC siRNA	negative control small interfering RNA

## References

[1] Burmester T., Weich B., Reinhardt S., Hankeln T. (2000). A vertebrate globin expressed in the brain. Nature.

[2] Wang X., Liu J., Zhu H., Tejima E., Tsuji K., Murata Y., Atochin D.N., Huang P.L., Zhang C., Lo E.H. (2008). Effects of neuroglobin overexpression on acute brain injury and long-term outcomes after focal cerebral ischemia. Stroke.

[3] Zhao S., Yu Z., Zhao G., Xing C., Hayakawa K., Whalen M.J., Lok J.M., Lo E.H., Wang X. (2012). Neuroglobin-overexpression reduces traumatic brain lesion size in mice. BMC Neurosci..

[4] Taylor J.M., Kelley B., Gregory E.J., Berman N.E. (2014). Neuroglobin overexpression improves sensorimotor outcomes in a mouse model of traumatic brain injury. Neurosci. Lett..

[5] Khan A.A., Mao X.O., Banwait S., Jin K., Greenberg D.A. (2007). Neuroglobin attenuates β-amyloid neurotoxicity in vitro and transgenic Alzheimer phenotype in vivo. Proc. Natl. Acad. Sci. USA.

[6] Li W., Wu Y., Ren C., Lu Y., Gao Y., Zheng X., Zhang C. (2011). The activity of recombinant human neuroglobin as an antioxidant and free radical scavenger. Proteins.

[7] Yu Z., Liu N., Li Y., Xu J., Wang X. (2013). Neuroglobin overexpression inhibits oxygen-glucose deprivation-induced mitochondrial permeability transition pore opening in primary cultured mouse cortical neurons. Neurobiol. Dis..

[8] Antao S.T., Duong T.T., Aran R., Witting P.K. (2010). Neuroglobin overexpression in cultured human neuronal cells protects against hydrogen peroxide insult via activating phosphoinositide-3 kinase and opening the mitochondrial K(ATP) channel. Antioxid. Redox Signal..

[9] Raychaudhuri S., Skommer J., Henty K., Birch N., Brittain T. (2010). Neuroglobin protects nerve cells from apoptosis by inhibiting the intrinsic pathway of cell death. Apoptosis.

[10] Yu Z., Liu N., Liu J., Yang K., Wang X. (2012). Neuroglobin, a novel target for endogenous neuroprotection against stroke and neurodegenerative disorders. Int. J. Mol. Sci..

[11] Wakasugi K., Nakano T., Morishima I. (2003). Oxidized human neuroglobin acts as a heterotrimeric Gα protein guanine nucleotide dissociation inhibitor. J. Biol. Chem..

[12] Khan A.A., Mao X.O., Banwait S., DerMardirossian C.M., Bokoch G.M., Jin K., Greenberg D.A. (2008). Regulation of hypoxic neuronal death signaling by neuroglobin. FASEB J..

[13] Li L., Liu Q.R., Xiong X.X., Liu J.M., Lai X.J., Cheng C., Pan F., Chen Y., Yu S.B., Yu A.C. (2014). Neuroglobin promotes neurite outgrowth via differential binding to PTEN and Akt. Mol. Neurobiol..

[14] Chen L.M., Xiong Y.S., Kong F.L., Qu M., Wang Q., Chen X.Q., Wang J.Z., Zhu L.Q. (2012). Neuroglobin attenuates Alzheimer-like tau hyperphosphorylation by activating Akt signaling. J. Neurochem..

[15] Li Y., Dai Y.B., Sun J.Y., Xiang Y., Yang J., Dai S.Y., Zhang X. (2016). Neuroglobin Attenuates Beta Amyloid-Induced Apoptosis Through Inhibiting Caspases Activity by Activating PI3K/Akt Signaling Pathway. J. Mol. Neurosci..

[16] Deng S., Ai Y., Gong H., Chen C., Peng Q., Huang L., Wu L., Zhang L., Zhang L. (2017). Neuroglobin Protects Rats from Sepsis-Associated Encephalopathy via a PI3K/Akt/Bax-Dependent Mechanism. J. Mol. Neurosci..

[17] Ascenzi P., di Masi A., Leboffe L., Fiocchetti M., Nuzzo M.T., Brunori M., Marino M. (2016). Neuroglobin: From structure to function in health and disease. Mol. Asp. Med..

[18] Palladino P., Scaglione G.L., Arcovito A., Maria Vitale R., Amodeo P., Vallone B., Brunori M., Benedetti E., Rossi F. (2011). Neuroglobin-prion protein interaction: What’s the function?. J. Pept. Sci..

[19] Wakasugi K., Nakano T., Morishima I. (2004). Association of human neuroglobin with cystatin C, a cysteine proteinase inhibitor. Biochemistry.

[20] Jayaraman T., Tejero J., Chen B.B., Blood A.B., Frizzell S., Shapiro C., Tiso M., Hood B.L., Wang X., Zhao X. (2011). 14-3-3 binding and phosphorylation of neuroglobin during hypoxia modulate six-to-five heme pocket coordination and rate of nitrite reduction to nitric oxide. J. Biol. Chem..

[21] De Marinis E., Fiocchetti M., Acconcia F., Ascenzi P., Marino M. (2013). Neuroglobin upregulation induced by 17*β*-estradiol sequesters cytocrome *c* in the mitochondria preventing H_2_O_2_-induced apoptosis of neuroblastoma cells. Cell Death Dis..

[22] Yu Z., Liu N., Wang Y., Li X., Wang X. (2012). Identification of Neuroglobin-Interacting Proteins Using Yeast Two-Hybrid Screening. Neuroscience.

[23] Deng N., Ye Y., Wang W., Li L. (2010). Dishevelled interacts with p65 and acts as a repressor of NF-κB-mediated transcription. Cell Res..

[24] Wharton K.A. (2003). Runnin’ with the Dvl: Proteins That Associate with Dsh/Dvl and Their Significance to Wnt Signal Transduction. Dev. Biol..

[25] Moon R.T., Shah K. (2002). Developmental biology: Signalling polarity. Nature.

[26] Sharma M., Castro-Piedras I., Simmons G.E., Pruitt K. (2018). Dishevelled: A masterful conductor of complex Wnt signals. Cell. Signal..

[27] Cai B., Li W., Mao X., Winters A., Ryou M.G., Liu R., Greenberg D.A., Wang N., Jin K., Yang S.H. (2016). Neuroglobin Overexpression Inhibits AMPK Signaling and Promotes Cell Anabolism. Mol. Neurobiol..

[28] Xiong X.X., Pan F., Chen R.Q., Hu D.X., Qiu X.Y., Li C.Y., Xie X.Q., Tian B., Chen X.Q. (2018). Neuroglobin boosts axon regeneration during ischemic reperfusion via p38 binding and activation depending on oxygen signal. Cell Death Dis..

[29] Gao C., Chen Y.G. (2010). Dishevelled: The hub of Wnt signaling. Cell Signal..

[30] Taelman V.F., Dobrowolski R., Plouhinec J.L., Fuentealba L.C., Vorwald P.P., Gumper I., Sabatini D.D., De Robertis E.M. (2010). Wnt signaling requires sequestration of glycogen synthase kinase 3 inside multivesicular endosomes. Cell.

[31] Peifer M., Polakis P. (2000). Wnt signaling in oncogenesis and embryogenesis—A look outside the nucleus. Science.

[32] Bielen H., Houart C. (2014). The Wnt cries many: Wnt regulation of neurogenesis through tissue patterning, proliferation, and asymmetric cell division. Dev. Neurobiol..

[33] Inestrosa N.C., Varela-Nallar L. (2015). Wnt signalling in neuronal differentiation and development. Cell Tissue Res..

[34] Huang T., Xie Z., Wang J., Li M., Jing N., Li L. (2011). Nuclear factor of activated T cells (NFAT) proteins repress canonical Wnt signaling via its interaction with Dishevelled (Dvl) protein and participate in regulating neural progenitor cell proliferation and differentiation. J. Biol. Chem..

[35] Haines B., Mao X., Xie L., Spusta S., Zeng X., Jin K., Greenberg D.A. (2013). Neuroglobin expression in neurogenesis. Neurosci. Lett..

[36] Luyckx E., Van Leuven W., Andre D., Quarta A., Reekmans K., Fransen E., Moens L., Hankeln T., Ponsaerts P., Dewilde S. (2018). Loss of Neuroglobin Expression Alters Cdkn1a/Cdk6-Expression Resulting in Increased Proliferation of Neural Stem Cells. Stem Cells Dev..

[37] Duckworth E.A.M., Butler T., Collier L., Collier S., Pennypacker K.R. (2006). NF-κB protects neurons from ischemic injury after middle cerebral artery occlusion in mice. Brain Res..

[38] Kaltschmidt B., Kaltschmidt C. (2010). NF-κB in the Nervous System (vol 1745, pg 287, 2005). Cold Spring Harbor Perspect. Biol..

[39] Schneider A., Martin-Villalba A., Weih F., Vogel J., Wirth T., Schwaninger M. (1999). NF-κB is activated and promotes cell death in focal cerebral ischemia. Nat. Med..

[40] Meffert M.K., Chang J.M., Wiltgen B.J., Fanselow M.S., Baltimore D. (2003). NF-κB functions in synaptic signaling and behavior. Nat. Neurosci..

[41] Yu Z., Zhou D., Bruce-Keller A.J., Kindy M.S., Mattson M.P. (1999). Lack of the p50 subunit of nuclear factor-κB increases the vulnerability of hippocampal neurons to excitotoxic injury. J. Neurosci..

[42] Xu Q., Deng F., Xing Z., Wu Z., Cen B., Xu S., Zhao Z., Nepomuceno R., Bhuiyan M.I.H., Sun D. (2016). Long non-coding RNA *C2dat1* regulates CaMKII*δ* expression to promote neuronal survival through the NF-*κ*B signaling pathway following cerebral ischemia. Cell Death Dis..

[43] Wajant H., Pfizenmaier K., Scheurich P. (2003). Tumor necrosis factor signaling. Cell Death Differ..

[44] Li X., Chen C., Wang F., Huang W., Liang Z., Xiao Y., Wei K., Wan Z., Hu X., Xiang S. (2014). KCTD1 suppresses canonical Wnt signaling pathway by enhancing β-catenin degradation. PLoS ONE.

